# First Isolation, Identification, and Whole-Genome Sequencing of a Multidrug-Resistant Bovine-Derived *Providencia stuartii* in China

**DOI:** 10.3390/pathogens15080789

**Published:** 2026-07-24

**Authors:** Rong-Jun Gong, Xue-Li Ge, Jia-Min Ma, Yang-Sini Fu, Jia-Hao Chen, Man-Ting Li, Yong-Xiang Zhao, Liang Zhu, Qing-Hong Guo, Xin-Chao Liu, Wen-Chao Li

**Affiliations:** 1 Anhui Province Key Laboratory of Animal Nutritional Regulation and Health, College of Animal Science, Anhui Science and Technology University, Chuzhou 233100, China; gongrongjun@ahstu.edu.cn (R.-J.G.);; 2 Agriculture and Rural Affairs Bureau of Panji District, Huainan 232082, China

**Keywords:** *Providencia stuartii*, cattle, whole-genome sequencing, multidrug resistance, virulence, pathogenicity

## Abstract

Background: *Providencia stuartii* is an opportunistic pathogen associated with multidrug resistance. However, its occurrence, genomic characteristics, antimicrobial resistance profiles, and pathogenic potential in cattle-associated isolates remain poorly understood. Methods: A *P. stuartii* strain was isolated from a rectal swab of a diarrheic beef cattle individual during bacterial investigation on a commercial farm in Anhui Province, China. The strain was identified by Gram staining, biochemical tests, 16S rRNA sequencing, and whole-genome sequencing. Antimicrobial susceptibility was assessed using the Kirby–Bauer method, and resistance and virulence genes were detected by PCR and genomic analysis. Pathogenicity was evaluated in a murine infection model. Results: The isolate was confirmed as *P. stuartii*, with a 4.22 Mb genome and 41.28% GC content. It showed multidrug resistance, including resistance to eight antimicrobial agents. Genomic analysis revealed multiple resistance and virulence determinants, genomic islands, prophages, and CRISPR regions. Under high-dose intraperitoneal challenge conditions, the isolate caused dose-dependent mortality, systemic recovery from major organs, and histopathological lesions in mice. Conclusions: This study reports the genomic and antimicrobial resistance characteristics of a multidrug-resistant *P. stuartii* strain recovered from a rectal swab of diarrheic beef cattle in China. High-dose intraperitoneal inoculation demonstrated that the isolate possessed pathogenic potential in mice under experimental conditions. However, the murine model did not reproduce natural exposure in cattle, and the findings do not establish bovine tissue invasion, clinical pathogenicity, or a causal role in diarrhea. Further epidemiological investigations and cattle-relevant experimental studies are required to determine the clinical significance of this organism in bovine populations.

## 1. Introduction

*Providencia stuartii* is an opportunistic pathogen belonging to the genus *Providencia* within the family *Enterobacteriaceae*. It is widely distributed in environmental reservoirs, including soil and water, as well as in the intestinal tracts of humans and animals [1]. Although generally considered a low-virulence organism, *P. stuartii* can cause a variety of healthcare-associated infections, particularly in immunocompromised individuals, patients with long-term indwelling catheters, and those exposed to prolonged broad-spectrum antimicrobial therapy. Clinical manifestations include urinary tract infections, septicemia, infective endocarditis, and pulmonary abscesses, and many clinical isolates exhibit multidrug-resistant phenotypes [2,3]. In addition to its intrinsic resistance to several antimicrobial agents, *P. stuartii* can acquire resistance determinants through mobile genetic elements, thereby limiting therapeutic options and facilitating the dissemination of antimicrobial resistance among bacterial populations.

Of particular concern is the ability of *P. stuartii* to produce extended-spectrum β-lactamases (ESBLs). In China, Chen et al. first reported a clinical *P. stuartii* isolate harboring the *bla*_CTX-M-14_ gene, highlighting the potential role of this species as a reservoir and disseminator of clinically important resistance determinants [4]. The emergence of multidrug-resistant and carbapenemase-producing *P. stuartii* strains has further increased concern regarding its clinical and public health significance. Recent genomic investigations have demonstrated that multidrug-resistant *P. stuartii* lineages and resistance-associated plasmids may spread between institutions and geographical regions, indicating that this organism should be considered an emerging antimicrobial resistance threat rather than solely an uncommon opportunistic pathogen.

Antimicrobial resistance is not confined to human healthcare settings but represents a complex challenge at the interface of humans, animals, food production systems, and the environment. The One Health concept recognizes that the health of humans, domestic and wild animals, plants, and ecosystems is closely interconnected. Accordingly, the control of antimicrobial resistance requires coordinated surveillance and intervention across human medicine, veterinary medicine, agriculture, and environmental health. The FAO, UNEP, WHO, and WOAH have identified antimicrobial resistance as a major One Health priority and have emphasized the need to monitor resistant bacteria and resistance determinants at the human–animal–environment interface (One Health Joint Plan of Action, 2022–2026) [5].

Food-producing animals are important components of this interface because their intestinal microbiota may harbor antimicrobial-resistant pathogenic and commensal bacteria. These organisms and their resistance genes may subsequently spread through direct animal contact, manure, contaminated water, farm environments, slaughter and food-processing chains, or animal-derived foods. Consequently, antimicrobial resistance in livestock bacteria is relevant not only to animal disease treatment and production efficiency but also to food safety and public health. The veterinary sector therefore plays a critical role in antimicrobial stewardship, laboratory diagnosis, resistance surveillance, and the early identification of emerging resistance mechanisms. Surveillance of less frequently investigated opportunistic bacteria, including *P. stuartii*, may reveal previously unrecognized reservoirs of clinically important resistance determinants.

Integrons are genetic platforms that capture, rearrange, and express gene cassettes and are important contributors to the development and dissemination of antimicrobial resistance. Barlow et al. isolated class 1- and class 2-integron-containing bacteria from bovine fecal samples without prior antibiotic selection [6]. Their study identified integron-containing bacteria from several genera, including urea-positive *P. stuartii*, and demonstrated that resistance-associated integrons may be present even when bacterial isolation is not performed under direct antimicrobial selective pressure. This finding is important because resistance genes that are weakly expressed or phenotypically silent in one bacterial host may remain undetected by conventional antibiotic-selection methods but could subsequently be transferred to other bacteria in which they are expressed.

Subsequently, Barlow and Gobius identified diverse class 2 integrons in bacteria originating from beef cattle sources [7]. Notably, they characterized a novel class 2 integron in *P. stuartii* that contained an apparently functional integrase but lacked the conventional antimicrobial resistance gene cassette array commonly associated with Tn7-like class 2 integrons. This observation suggested that bovine-associated *P. stuartii* may harbor unusual and potentially evolutionarily important integron structures. Together, these studies indicate that cattle-associated bacterial communities can serve as reservoirs of diverse mobile genetic elements and support the inclusion of livestock-derived *P. stuartii* in antimicrobial resistance investigations conducted within a One Health framework.

Compared with the extensive literature available in human medicine, reports of *P. stuartii* in animals remain relatively scarce. Waldhalm et al. were the first to isolate *P. stuartii* from diarrheic calves, demonstrating its potential involvement in disease in cattle [8]. Subsequently, Liu et al. described a fatal case of septicemia caused by *P. stuartii* in an Indian rhesus macaque (*Macaca mulatta*), in which severe necrotizing suppurative meningoencephalitis and bronchopneumonia were observed during pathological examination [9]. Furthermore, Kurmasheva et al. demonstrated using an in vitro cell culture model that clinical isolates of *P. stuartii* possess motility, adhesion, and invasion capabilities toward HeLa cells, with invasion efficiency influenced by both bacterial growth phase and multiplicity of infection [10]. These findings collectively indicate that *P. stuartii* possesses pathogenic potential in animal hosts and may contribute to disease development under appropriate conditions. Nevertheless, information regarding the biological characteristics, genomic features, antimicrobial resistance mechanisms, and pathogenicity of bovine-derived *P. stuartii* remains limited.

In the present study, a *P. stuartii* strain was isolated from a rectal swab collected from beef cattle on a commercial farm in Anhui Province, China. The isolate was comprehensively characterized through phenotypic identification, antimicrobial susceptibility testing, whole-genome sequencing, virulence gene profiling, and pathogenicity evaluation in a mouse model. To the best of our knowledge, this is the first report of *P. stuartii* isolated from beef cattle feces in China. This study provides insights into the biological characteristics, pathogenic potential, and antimicrobial resistance profile of bovine-derived *P. stuartii*. The findings may contribute to a better understanding of the genomic characteristics, antimicrobial resistance mechanisms, and pathogenic potential of cattle-associated *P. stuartii*. However, because the isolate was obtained from a diarrheic animal rather than from confirmed lesions attributable to *P. stuartii*, its direct role in bovine diarrhea requires further investigation.

## 2. Materials and Methods

### 2.1. Sample Collection

Rectal swab samples were collected from beef cattle exhibiting diarrhea on a commercial farm in Anhui Province, China. The affected animals showed clinical signs including loose feces or diarrhea at the time of sampling. Samples were collected for bacterial investigation to identify potential enteric pathogens. The swabs were placed into sterile microcentrifuge (EP) tubes immediately after collection and transported to the laboratory under refrigerated conditions in insulated containers containing ice packs for subsequent bacteriological analysis.

### 2.2. Bacterial Isolation and Culture

Under aseptic conditions, each rectal swab sample was suspended in sterile physiological saline and thoroughly mixed. The resulting suspension was streaked onto MacConkey agar and tryptic soy agar (TSA) plates (Qingdao Hope Bio-Technology Co., Ltd., Qingdao, China) using the standard streak plate method. Plates were incubated aerobically at 37 °C for 24 h.

Following incubation, morphologically similar dominant colonies were selected and subcultured three consecutive times to obtain pure isolates. A single purified colony was then subjected to Gram staining, and bacterial morphology was examined under a light microscope. Biochemical characteristics of the isolate were subsequently determined using a commercial biochemical identification system (Qingdao Hope Bio-Technology Co., Ltd., Qingdao, China) according to the manufacturer’s instructions.

### 2.3. Identification of the Isolate by 16S rDNA Sequencing

Genomic DNA was prepared according to the method described by Cao et al. [11] and used as the template for PCR amplification. Two pairs of universal bacterial primers were employed to amplify the 16S rDNA gene. Each PCR reaction was performed in a total volume of 50 μL, consisting of 25 μL of PCR Master Mix, 1 μL each of the forward and reverse primers, 2 μL of DNA template, and nuclease-free double-distilled water (ddH_2_O) to the final volume.

The PCR amplification program was as follows: an initial denaturation at 94 °C for 5 min, followed by 30 cycles of denaturation at 94 °C for 30 s, annealing at 55 °C for 30 s, and extension at 72 °C for 90 s, with a final extension at 72 °C for 7 min.

The amplified products were analyzed by electrophoresis on a 1.0% agarose gel and subsequently purified and sequenced by Sangon Biotech Co., Ltd. (Shanghai, China). The obtained sequences were compared against reference sequences available in the National Center for Biotechnology Information (NCBI) database using the NCBI BLAST program (https://blast.ncbi.nlm.nih.gov/Blast.cgi, accessed on 20 April 2026). Phylogenetic relationships were further inferred using the Neighbor-Joining (NJ) method implemented in MEGA version 11.0 software.

### 2.4. Whole-Genome Sequencing and Bioinformatic Analysis

A single colony was selected from the TSA plate and cultured in 5 mL of sterile brain heart infusion (BHI) broth at 37 °C with shaking at 200 rpm for 16 h. The overnight culture was subsequently diluted 1:100 into 200 mL of fresh BHI broth and incubated under the same conditions for 4 h. The bacterial cells were harvested by centrifugation (model H3-18K, Hunan Course Instrument Equipment Co., Ltd., China) at 4 °C and 4000× *g* for 10 min after transferring the culture into sterile 50 mL centrifuge tubes. The supernatant was discarded, and the cell pellets were washed twice with sterile phosphate-buffered saline (PBS; Macklin Biochemical Co., Ltd., Shanghai, China) using the same centrifugation conditions to remove residual medium components and extracellular contaminants. The final bacterial suspension was prepared by resuspending the washed cells in bacterial preservation solution.

Bacterial genomic DNA of the *P. stuartii* isolate was submitted to Anhui General Biotech Co., Ltd. (Hefei, China) for whole-genome sequencing using the Illumina NovaSeq platform (Illumina Inc., San Diego, CA, USA) with paired-end reads. Raw sequencing reads were quality-filtered and adapter-trimmed using fastp (v0.23.2), followed by *de novo* genome assembly using SPAdes (v3.15.5). The assembled genome was subsequently polished using Pilon (v1.24).

Genome visualization was performed using Circos (v0.69-9). Protein-coding genes were predicted using GeneMarkS (v4.32) and functionally annotated against the NCBI non-redundant protein (NR), eggNOG (v5.0), Gene Ontology (GO), Swiss-Prot, and Kyoto Encyclopedia of Genes and Genomes (KEGG) databases. Virulence-associated genes were identified using the Virulence Factor Database (VFDB) and the Pathogen–Host Interactions database (PHI-base, v4.16), whereas antimicrobial resistance genes were predicted using the Comprehensive Antibiotic Resistance Database (CARD, v3.2.9) and ResFinder (v4.1). Genomic islands and integrons were identified using IslandViewer 4 and IntegronFinder (v2.0), respectively.

### 2.5. Antimicrobial Susceptibility Testing

The isolate was initially recovered and purified on tryptic soy agar (TSA) at 37 °C for 24 h. A well-isolated colony was inoculated into brain heart infusion (BHI) broth (Qingdao Hope Bio-Technology Co., Ltd., Qingdao, China) and incubated at 37 °C with shaking at 200 rpm for 24 h. Following incubation, the bacterial suspension was adjusted with sterile saline to a turbidity equivalent to a 0.5 McFarland standard.

Disk diffusion testing was performed on Mueller–Hinton agar (MHA) (Qingdao Hope Bio-Technology Co., Ltd., Qingdao, China) in accordance with the standardized procedure described in CLSI M02, 14th edition [12]. The standardized suspension was evenly inoculated over the entire MHA surface using a sterile swab. Antimicrobial disks (Hangzhou Microbial Reagent Co., Ltd., Hangzhou, China) were applied to the inoculated plates, which were subsequently incubated aerobically at 37 °C for 24 h. The diameters of the inhibition zones were measured to the nearest whole millimeter.

CLSI M100, 34th edition, was consulted for interpretive criteria. However, validated species-specific disk diffusion breakpoints are not available for *Providencia stuartii* for all of the antimicrobial agents included in this study [13]. Therefore, inhibition-zone diameters are reported as quantitative measurements without susceptible, intermediate, or resistant categorical interpretation for agents lacking validated criteria.

### 2.6. Determination of the Growth Curve of the Isolate

The preserved *P. stuartii* isolate was inoculated into 5 mL of Luria–Bertani (LB) broth (Qingdao Hope Bio-Technology Co., Ltd., Qingdao, China) and incubated overnight at 37 °C with shaking. Subsequently, 5 mL of the overnight culture was transferred into 100 mL of sterile LB broth and cultured under the same conditions. Samples were collected at 2 h intervals, and bacterial growth was monitored by measuring the optical density at 600 nm (OD_600_) using a UV–visible spectrophotometer (722N, Shanghai Yidian Analytical Instrument Co., Ltd., Shanghai, China). Measurements were performed continuously for 24 h. The obtained OD_600_ values were used to construct the bacterial growth curve and evaluate the growth characteristics of the isolate.

### 2.7. Detection of Virulence-Associated Genes

Based on a previous report [14], six major virulence-associated genes commonly detected in *P. stuartii* were selected for PCR screening. The primer sequences used in this study are listed in Table 1. Primers were synthesized by Sangon Biotech Co., Ltd. (Shanghai, China).

PCR amplification was performed in a total reaction volume of 25 μL, containing 6.5 μL of PCR Master Mix (TaKaRa Bio Inc., Shiga, Japan), 0.5 μL each of the forward and reverse primers, 2 μL of template DNA, and nuclease-free double-distilled water (ddH_2_O) added to the final volume.

The amplification protocol consisted of an initial denaturation at 94 °C for 5 min, followed by 30–35 cycles of denaturation at 94 °C for 30 s, annealing at 55 °C for 30 s, and extension at 72 °C for 30–60 s, depending on the expected amplicon size. A final extension step was performed at 72 °C for 5 min. Amplified products were subsequently analyzed by agarose gel electrophoresis.

### 2.8. Detection of Antimicrobial Resistance Genes

To investigate the genetic basis of antimicrobial resistance, seven resistance-associated genes commonly reported in pathogenic Escherichia coli were selected according to a previously published method [15]. The primer sequences are presented in Table 2.

PCR amplification was carried out in a total volume of 25 μL containing 12.5 μL of PCR Master Mix, 1 μL each of the forward and reverse primers, 2 μL of template DNA, and nuclease-free double-distilled water (ddH_2_O) to the final volume.

The thermal cycling conditions were as follows: initial denaturation at 94 °C for 5 min; 35 cycles of denaturation at 94 °C for 30 s, annealing at 57 °C for 30 s, and extension at 72 °C for 1 min; followed by a final extension at 72 °C for 10 min. PCR products were analyzed by agarose gel electrophoresis to determine the presence of the target resistance genes.

### 2.9. Pathogenicity Test of the Isolate in Mice

The murine intraperitoneal challenge model was used as a preliminary in vivo assay to assess whether the isolate could survive, disseminate, and induce host injury under controlled experimental conditions. This model was not intended to reproduce the natural route of intestinal exposure in cattle or to establish the isolate as a causative agent of bovine diarrhea. Therefore, the findings obtained from this model were interpreted only as evidence of experimental pathogenic potential in mice.

#### 2.9.1. Determination of the Median Lethal Dose (LD_50_)

The *P. stuartii* isolate was cultured in brain heart infusion (BHI) broth at 37 °C with shaking for 18 h. The bacterial concentration was determined by serial tenfold dilution and plate counting on TSA. Dilutions yielding 30–300 colonies were selected for enumeration.

A total of 48 male ICR mice (approximately 20 g body weight) were randomly assigned to six groups (*n* = 8 per group). Five bacterial inocula (10^10^, 10^9^, 10^8^, 10^7^, and 10^6^ CFU/mL) were prepared by serial dilution or concentration of the original culture. Mice were intraperitoneally inoculated with 0.4 mL of bacterial suspension, while control mice received sterile saline. Clinical signs and mortality were monitored for 7 days post-infection. The LD_50_ value was calculated using the Reed–Muench method based on cumulative mortality.

#### 2.9.2. Histopathological Examination

At 7 days post-infection, mice were euthanized, and liver, spleen, and kidney tissues were collected and fixed in formalin for one week. Histological sections were prepared according to a previously described protocol [16] and examined microscopically for pathological changes.

#### 2.9.3. Quantification of Bacterial Loads in Organs

Twelve male ICR mice (approximately 20 g body weight) were randomly divided into two groups (*n* = 6 per group). Following intraperitoneal inoculation with the bacterial suspension, mice that succumbed to infection within 24 h were necropsied. Liver, spleen, and kidney tissues (30 mg each) were collected and homogenized in 270 μL of sterile phosphate-buffered saline (PBS) using a tissue homogenizer (Tiss-Basic48, Shanghai Jingxin Industrial Development Co., Ltd., Shanghai, China) with sterile grinding beads at 60 Hz for 5 min. 

The homogenates were serially diluted (10^−4^–10^−6^), and 100 μL of each dilution was plated onto TSA in duplicate. After incubation at 37 °C for 24 h, colonies were counted, and bacterial loads were expressed as CFU per gram of tissue.

## 3. Results

### 3.1. Cultural Characteristics of the P. stuartii Isolate

**Figure 1:**
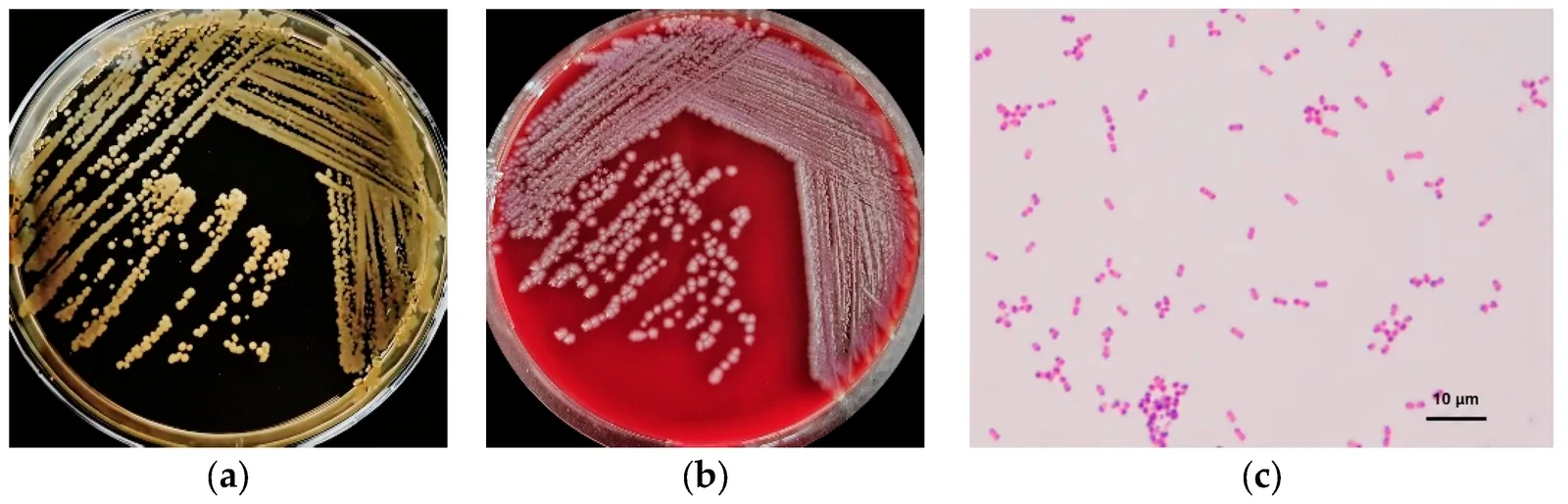
Colony morphology of the *P. stuartii* isolate (**a**) Colonial morphology of *P. stuartii* cultured on TSA medium; (**b**) Colonial morphology of *P. stuartii* cultured on blood agar plates; (**c**) Microscopic morphology of *P. stuartii* after Gram staining.

The isolate produced medium-sized, creamy-white, circular colonies with smooth margins, convex centers, and a moist, glossy surface on TSA (Figure 1a). No swarming motility was observed. On blood agar, the isolate formed grayish-white, smooth, and moist colonies with regular margins (Figure 1b), without hemolysis or swarming growth.

Microscopic examination under oil immersion revealed that the isolate was a Gram-negative short rod with rounded ends following Gram staining (Figure 1c).

### 3.2. Biochemical Characteristics

The isolated strain was subjected to biochemical identification following the manufacturer’s instructions for biochemical identification tubes, and the results are shown in Table 3.

### 3.3. Identification by 16S rDNA Sequencing

PCR amplification of the 16S rDNA gene yielded the expected product, which was purified and sequenced. BLAST analysis against the NCBI database demonstrated high sequence similarity with members of the genus *Providencia*, preliminarily identifying the isolate as *Providencia* spp.

**Figure 2:**
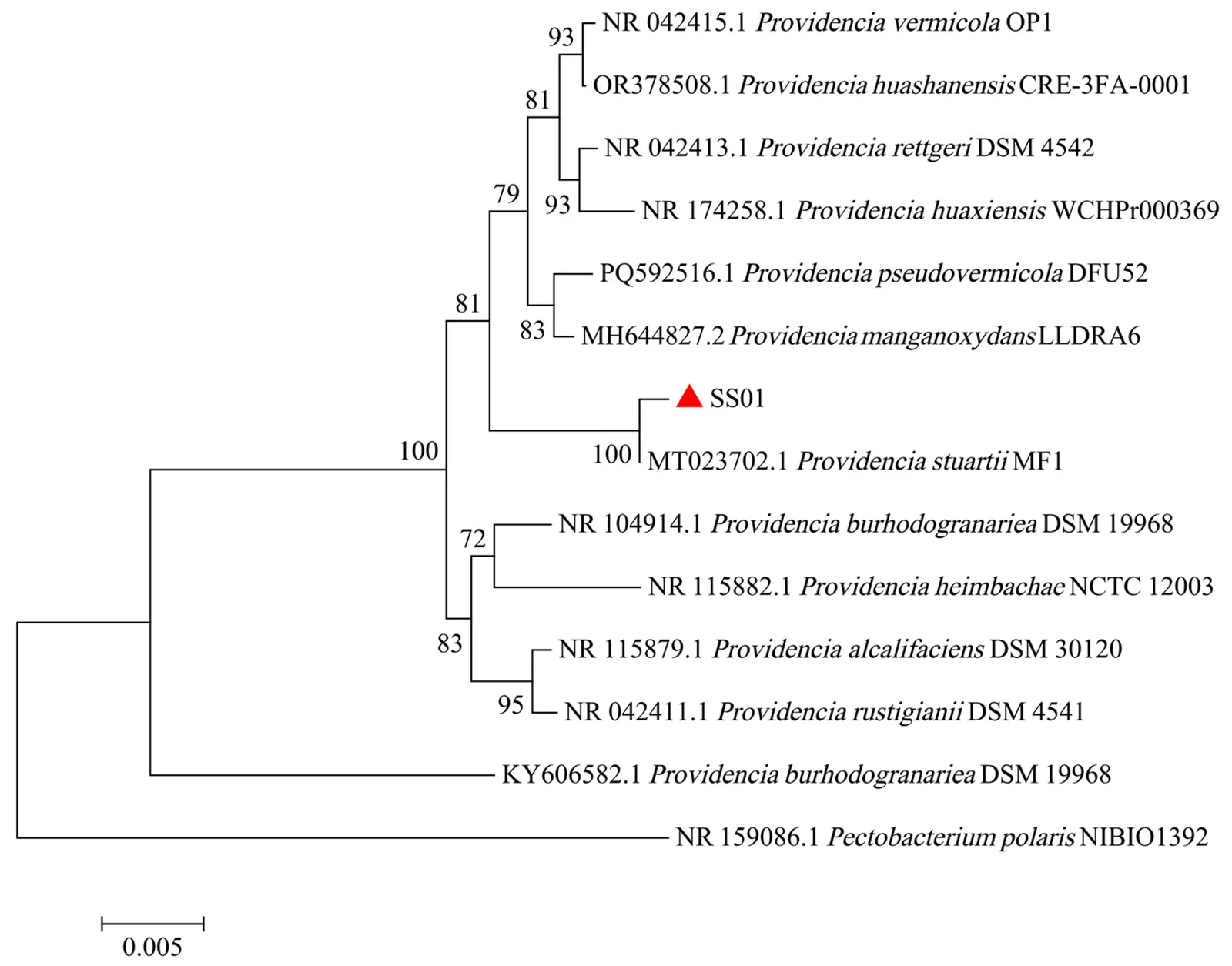
Phylogenetic tree of the isolated *P. stuartii* strain constructed based on the 16S rRNA gene. Red triangle SS01 is the isolate investigated in this study.

The 16S rRNA gene sequences of 13 related strains were obtained from the GenBank database. A phylogenetic tree was constructed using MEGA version 11.0 to analyze the evolutionary relationship between the bovine-derived *P. stuartii* isolate and closely related reference strains (Figure 2).

### 3.4. Whole-Genome Sequencing Analysis

Whole-genome sequence analysis showed 99% nucleotide identity with the reference *P. stuartii* strain CP048621.1. The genome sequence has been deposited in GenBank under accession number GCA_056897575.1.

**Figure 3:**
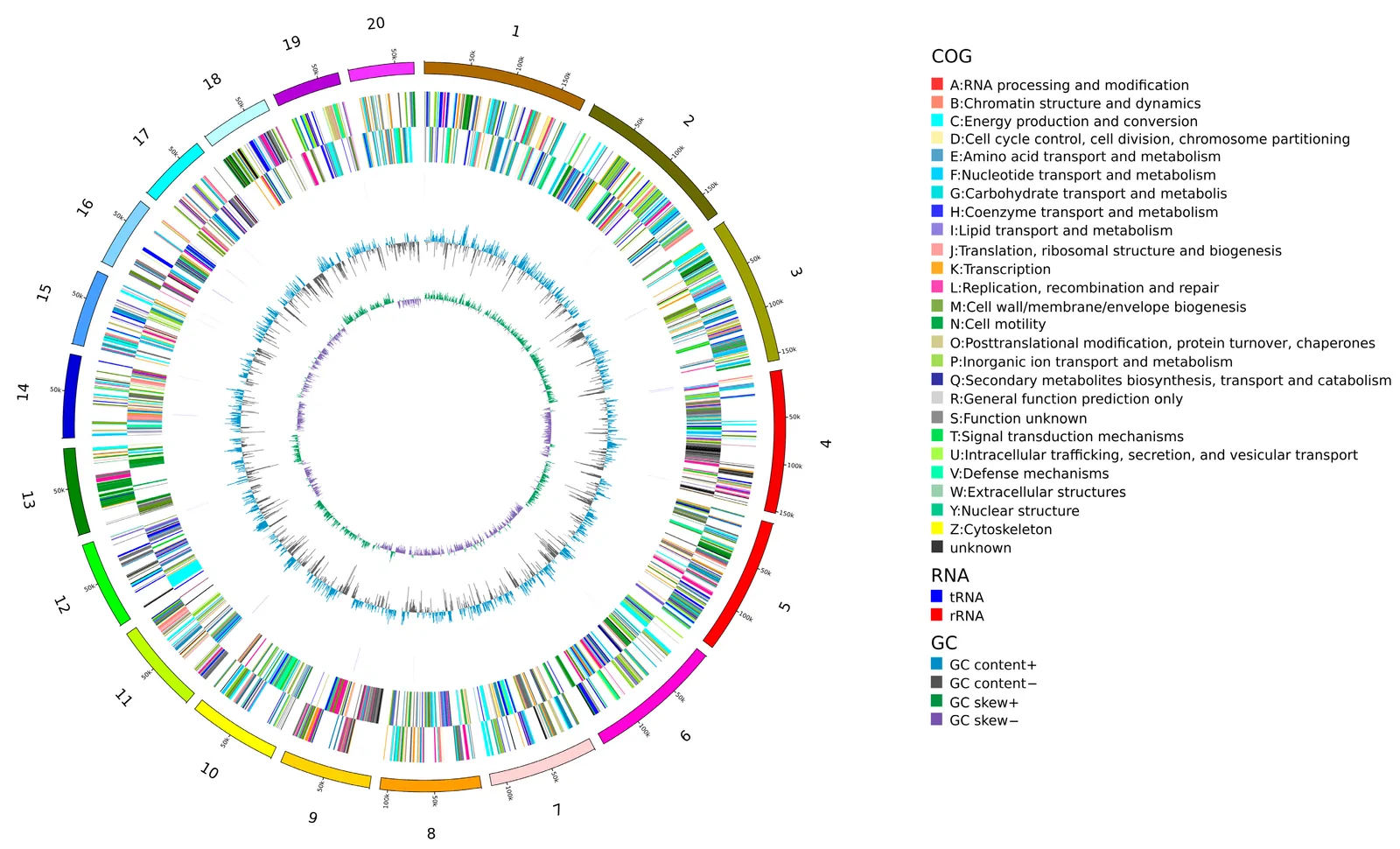
Circular mapping of the whole genome of the *P. stuartii* isolate.

The assembled genome consisted of 4,221,436 bp with a GC content of 41.3%, comprising 192 contigs and 179 scaffolds, including 150 sequences longer than 1 kb. A total of 3948 protein-coding genes were predicted (Figure 3).

In addition, four CRISPR regions, multiple prophage regions, and several genomic islands were identified. Analyses of transposable elements, insertion sequences, and repetitive sequences were also performed.

**Figure 4:**
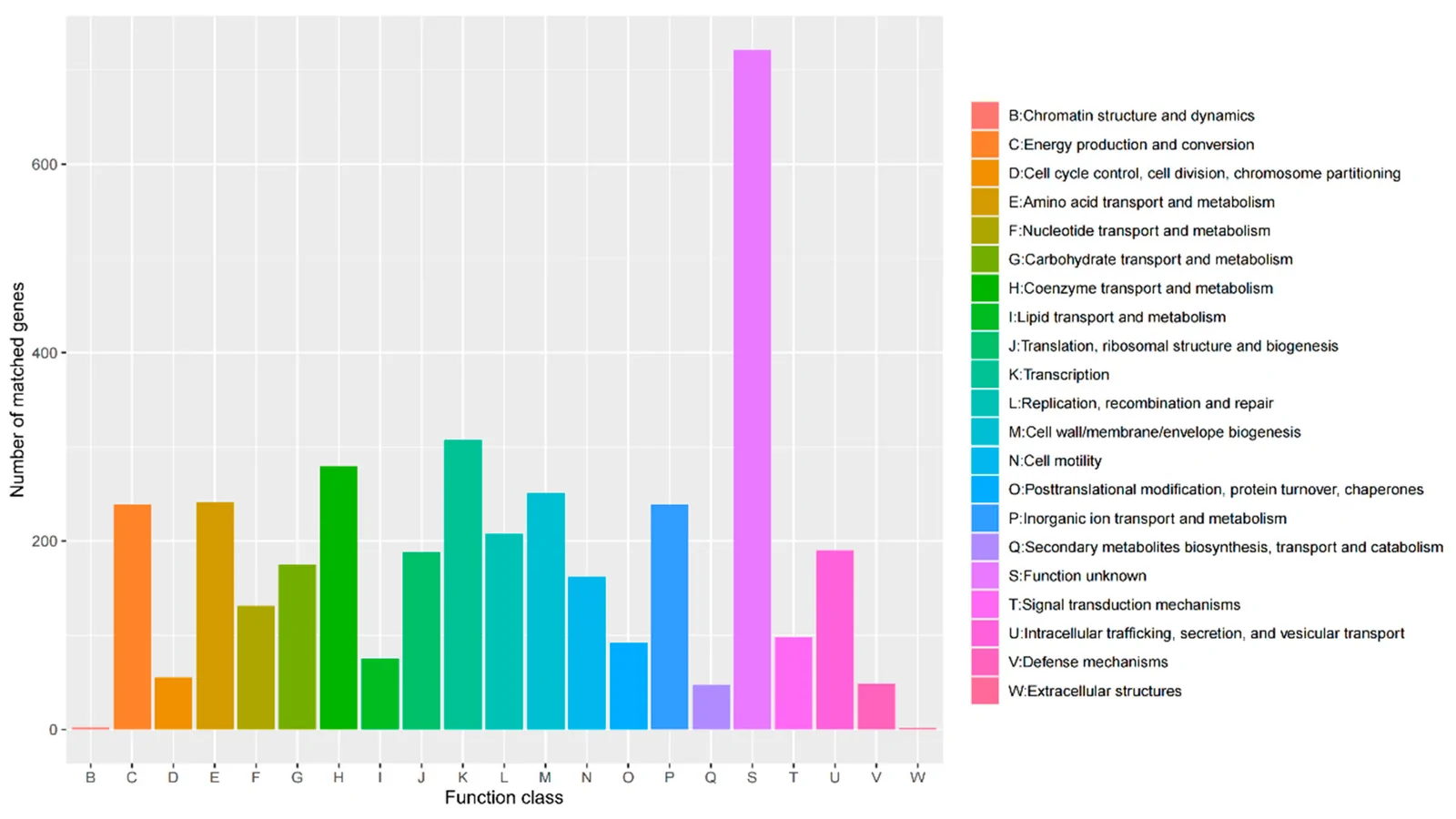
COG function classification of the *P. stuartii* isolate.

Functional annotation based on the COG, KEGG, and GO databases revealed that genes involved in transcription represented the largest functional category (307 genes), followed by genes associated with amino acid transport and metabolism, carbohydrate transport and metabolism, and coenzyme transport and metabolism (Figure 4).

**Figure 5:**
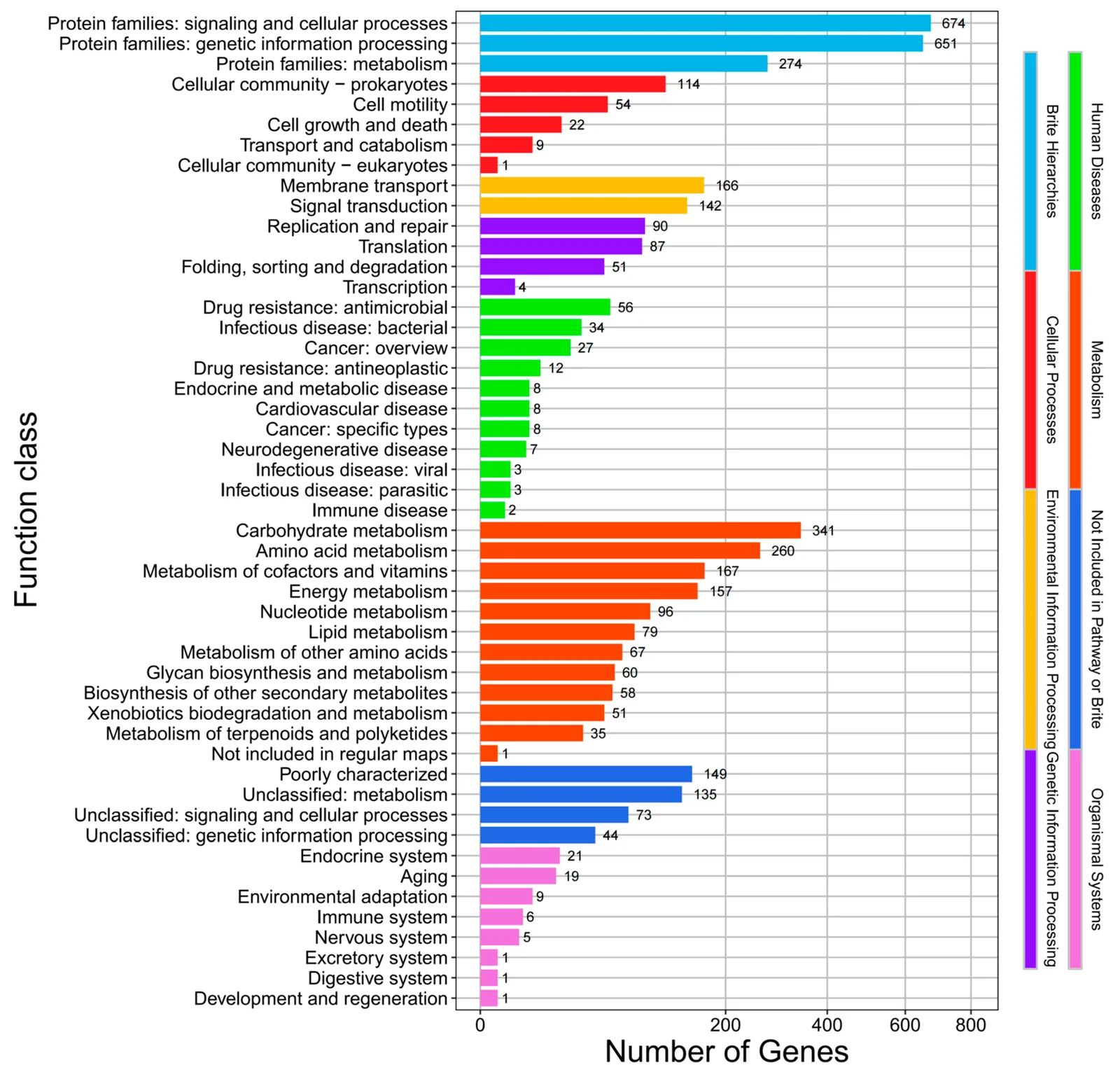
KEGG classification of the *P. stuartii* isolate.

KEGG pathway annotation revealed that genes associated with metabolic functions constituted the largest proportion of the genome. Among these, pathways involved in carbohydrate metabolism (341 genes), amino acid metabolism (260 genes), metabolism of cofactors and vitamins (167 genes), and energy metabolism (157 genes) were particularly abundant, indicating a strong metabolic capacity and environmental adaptability of the isolate. In addition to metabolism-related pathways, a substantial number of genes were assigned to signaling and cellular processes (674 genes) and genetic information processing (651 genes). Within these categories, genes associated with membrane transport (166 genes), signal transduction (142 genes), replication and repair (90 genes), and translation (87 genes) were prominently represented. Furthermore, annotation of genes related to human diseases identified 56 genes associated with antimicrobial drug resistance and 34 genes involved in bacterial infectious diseases, suggesting potential pathogenicity and resistance capabilities of the isolate (Figure 5).

**Figure 6:**
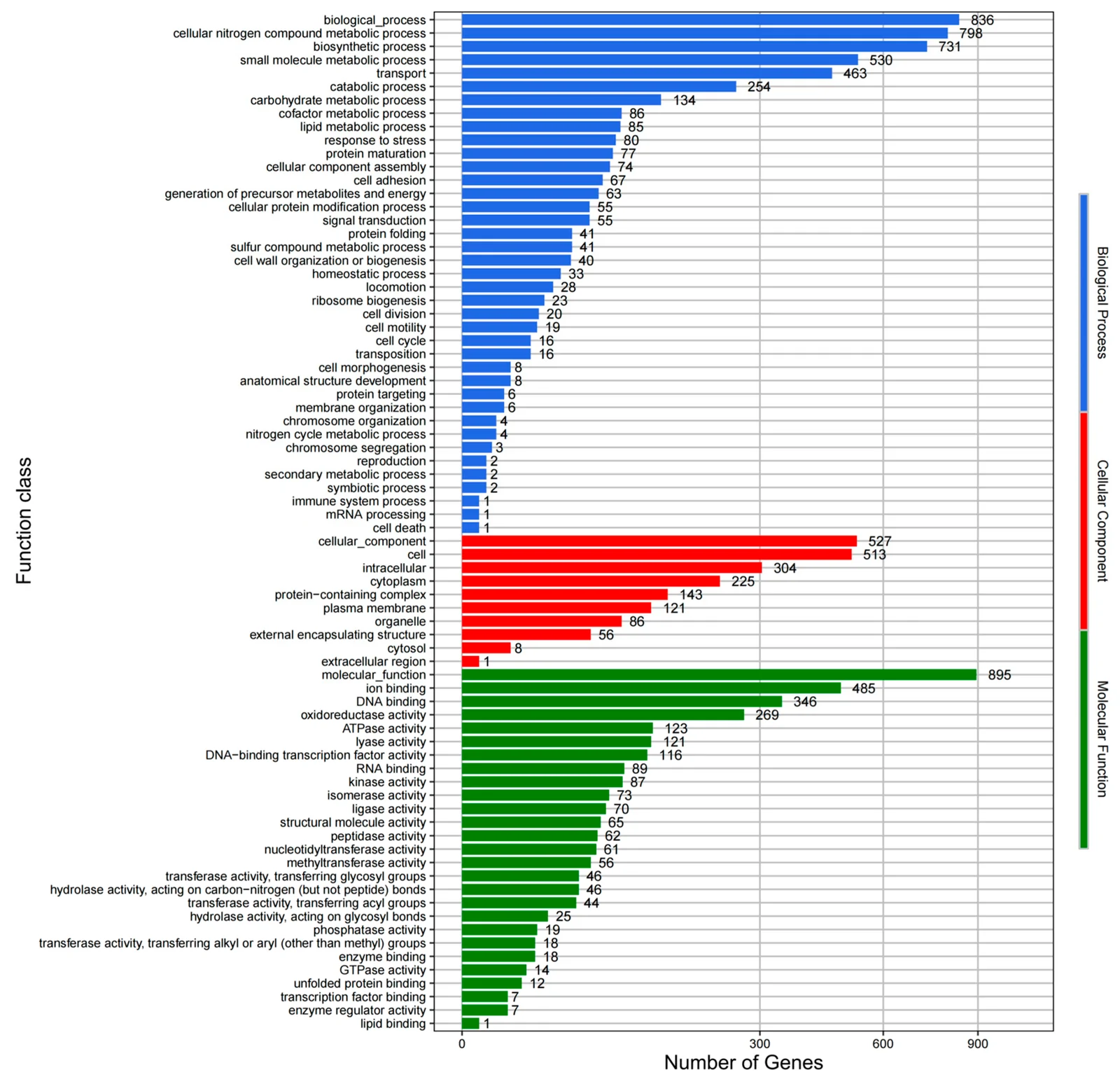
GO classification of the *P. stuartii* isolate.

Gene ontology (GO) classification analysis further characterized the functional composition of the genome. Within the biological process category, genes involved in fundamental metabolic activities and substance transport were predominant, including those associated with cellular nitrogen compound metabolic processes (798 genes), biosynthetic processes (731 genes), small-molecule metabolic processes (530 genes), and transport (463 genes). In the cellular component category, most gene products were predicted to be localized to the cell (513 genes), intracellular compartments (304 genes), and the cytoplasm (225 genes). The molecular function category was dominated by genes involved in ion binding (485 genes) and DNA binding (346 genes), highlighting the importance of molecular interactions and genetic regulation in the biological activities of the isolate (Figure 6).

Virulence factor prediction based on the virulence factor database (VFDB) identified a total of 121 virulence-associated genes within the genome. Among these, genes involved in immune modulation represented the largest functional category, comprising 37 genes (30.6% of all predicted virulence genes). This was followed by genes associated with motility (22 genes, 18.2%) and nutritional/metabolic factors (20 genes, 16.5%). Analysis using the comprehensive antibiotic resistance database (CARD) identified 113 antimicrobial resistance-related genes. The most abundant resistance mechanism was antibiotic efflux, accounting for 53 genes (46.9% of all resistance-associated genes), followed by antibiotic target alteration, represented by 36 genes (31.9%).

### 3.5. Antimicrobial Susceptibility Profile

The diameters of inhibition zones measured via the disk diffusion assay are summarized in Table 4. Validated species-specific interpretive breakpoints for *Providencia stuartii* are not available for a number of tested antimicrobials; accordingly, only raw zone diameters are listed for these agents without categorical susceptibility classification (susceptible, intermediate, resistant). Small inhibition zones were obtained against gentamicin, kanamycin, amikacin, tetracycline, minocycline and erythromycin, while large clear zones were observed for ceftazidime, ceftriaxone, cefoperazone, norfloxacin and ofloxacin. Although these phenotypic susceptibility profiles were analyzed alongside the identified resistance genes (*bla*_CTX-M_, *aad*A1, *sul*1 and *tet*A), formal resistance categorization was not performed due to the lack of official species-specific interpretive criteria.

### 3.6. Growth Characteristics of the Isolate

**Figure 7:**
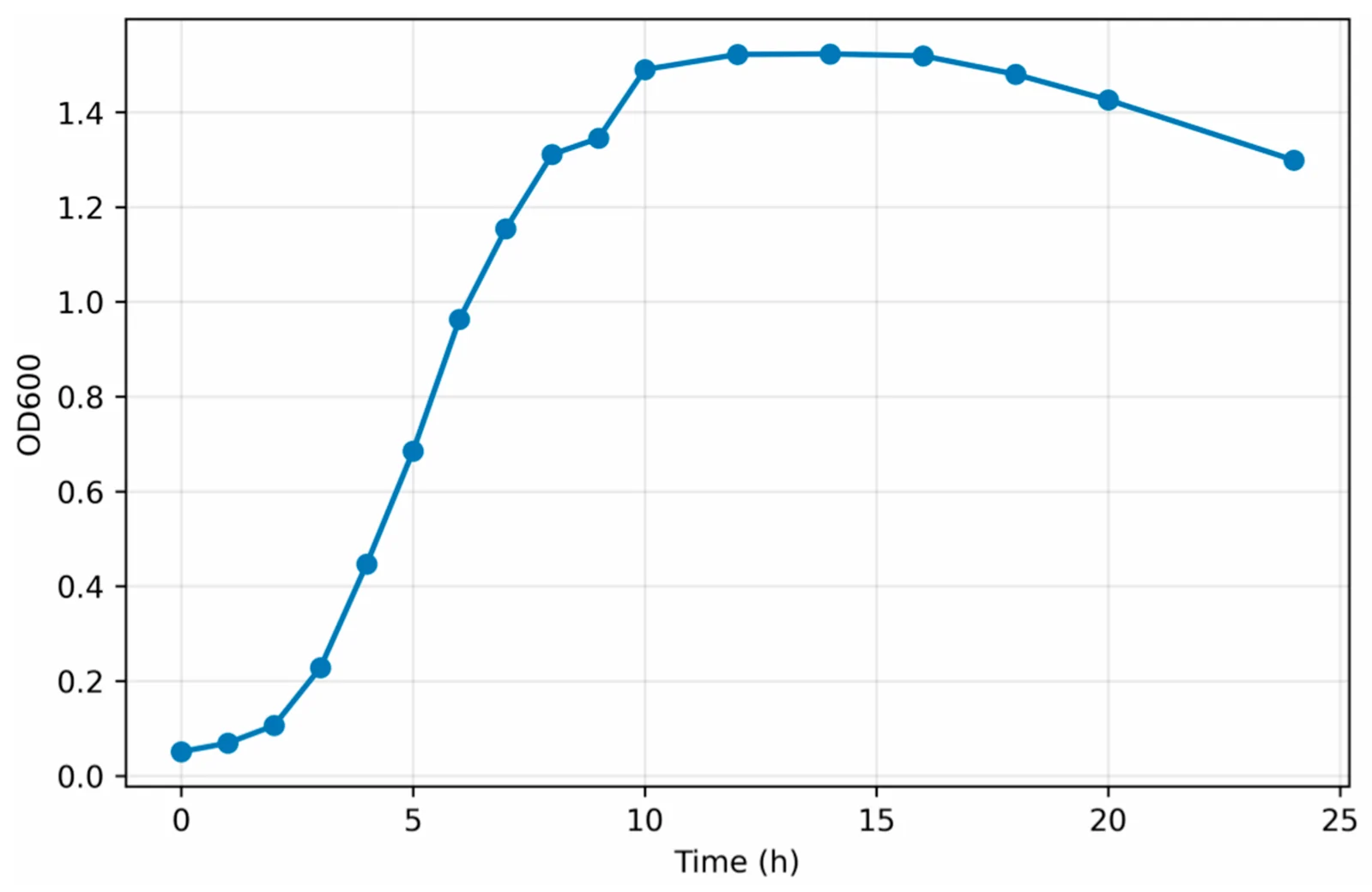
Growth curve of the *P. stuartii* isolate.

The growth curve of *P. stuartii* cultured in LB broth is shown in Figure 7. During the initial incubation period (0–2 h), the isolate remained in the lag phase, characterized by a slow increase in OD_600_ values. Thereafter, the bacteria entered the exponential growth phase between 4 and 12 h, during which rapid proliferation was observed, accompanied by a marked increase in OD_600_. After approximately 12 h of incubation, the growth rate gradually declined, and the culture entered the stationary phase. The OD_600_ values reached a maximum between 16 and 20 h and subsequently decreased, indicating the onset of the decline phase. These results demonstrated that *P. stuartii* exhibited a typical bacterial growth pattern in LB broth, with a distinct exponential growth phase occurring primarily between 4 and 12 h.

### 3.7. Detection Results of Virulence Genes

**Figure 8:**
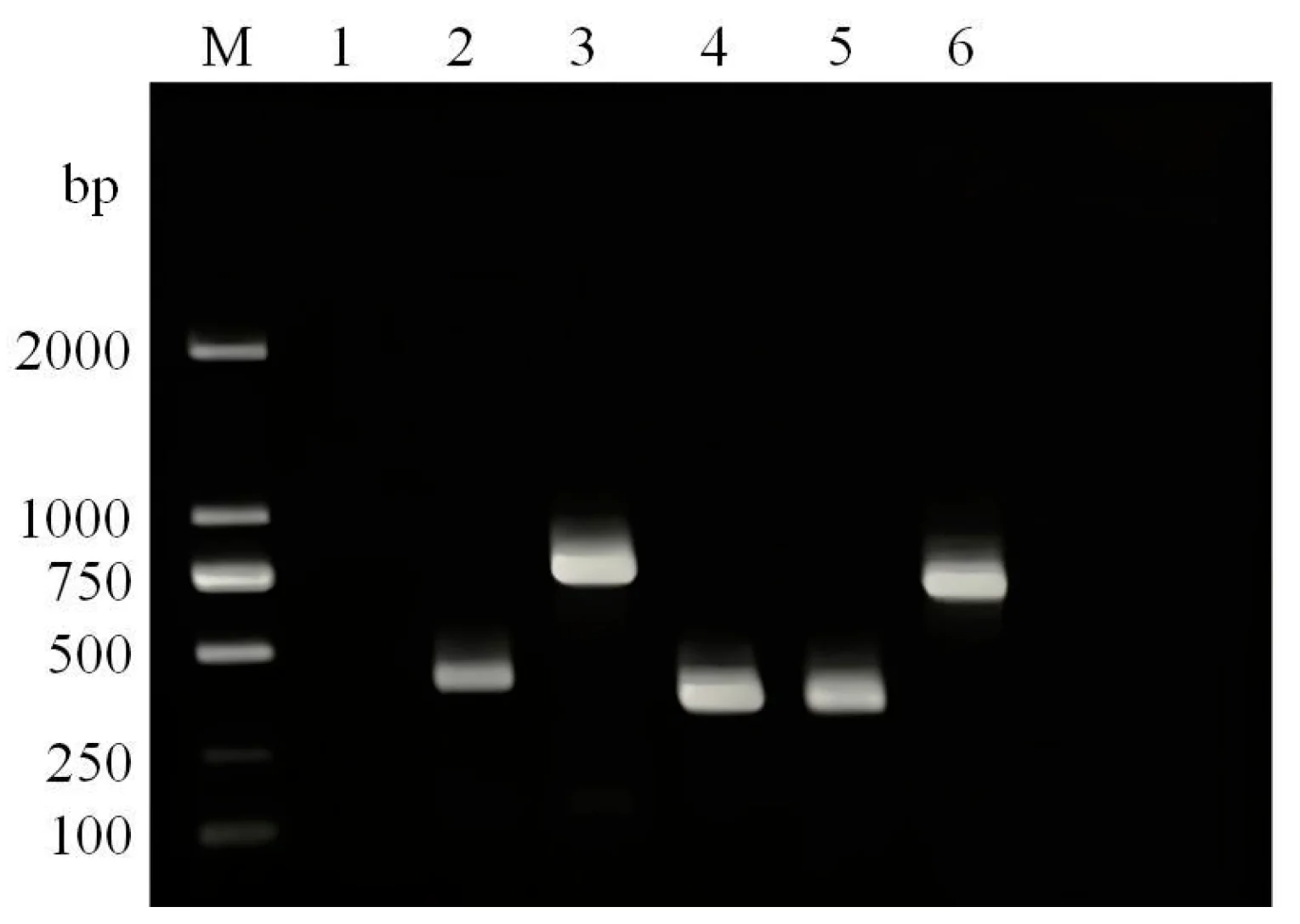
PCR results of virulence genes of the *P. stuartii* isolate M, DL2000 DNA Marker; 1–6, PCR amplification products of *fim*A, *mrk*A, *fpt*A, *ire*A, *iut*A and *hly*A genes, respectively.

As shown in Figure 8, PCR amplification successfully detected five virulence-associated genes in the isolate, namely *mrk*A (411 bp), *fpt*A (744 bp), *ire*A (351 bp), *iut*A (347 bp), and *hly*A (701 bp). The sizes of all amplified fragments were consistent with the expected target products. Overall, five of the six screened virulence genes were detected, yielding a detection rate of 83.3%.

### 3.8. Detection Results of Antimicrobial Resistance Genes

**Figure 9:**
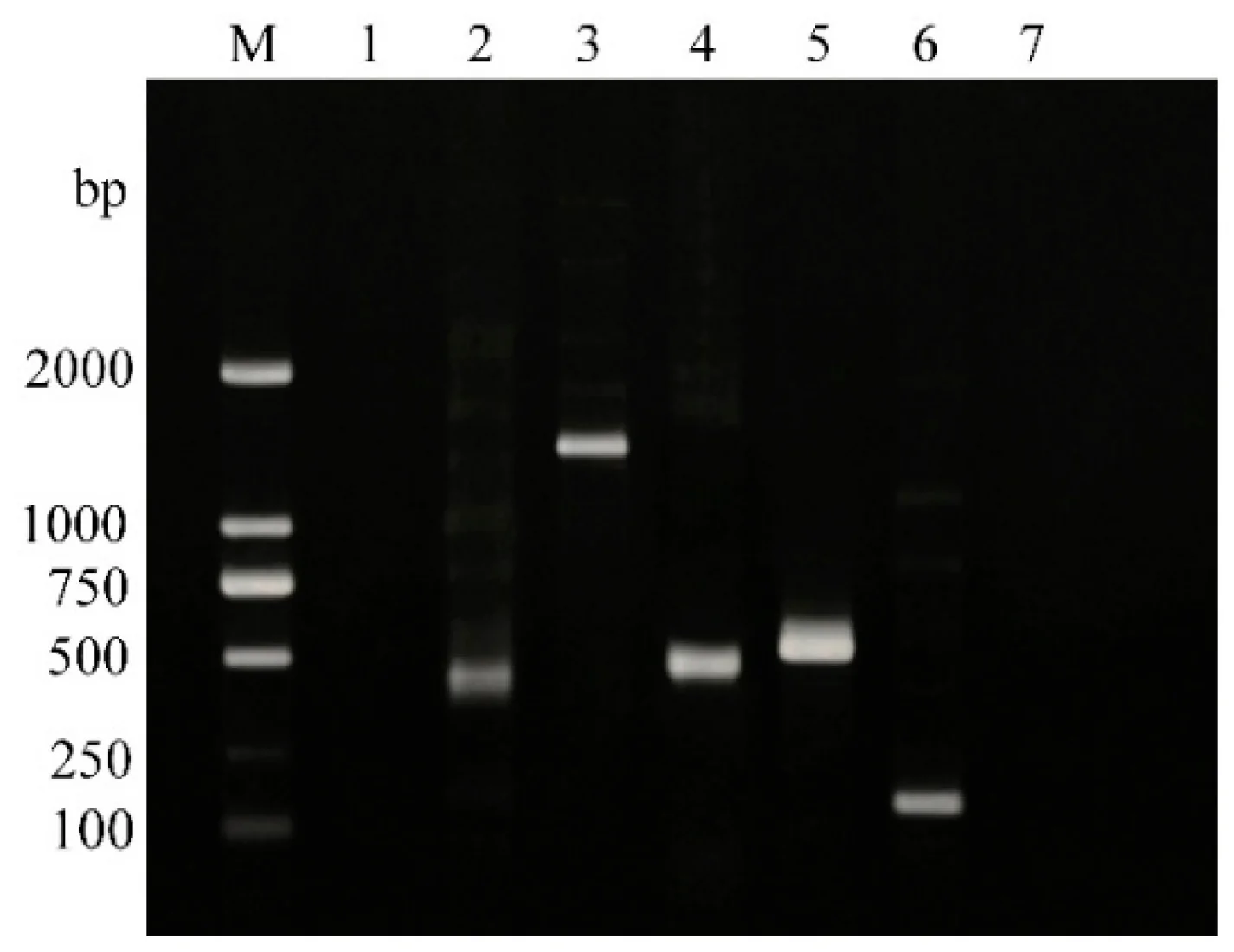
PCR results of antimicrobial resistance genes of the *P. stuartii* isolate M, DL2000 DNA Marker; 1–7, PCR amplification products of *bal*_TEM-1_, *bla*_CTX-M_, *aac*3, *aad*A1, *sul*1, *tet*A and cat genes, respectively.

As illustrated in Figure 9, four antimicrobial resistance genes were identified in the isolate. These included the β-lactam resistance gene *bla*_CTX-M_ (416 bp), the aminoglycoside resistance gene *aad*A1 (527 bp), the sulfonamide resistance gene *sul*1 (555 bp), and the tetracycline resistance gene *tet*A (175 bp). The sizes of the amplified PCR products corresponded precisely to the expected fragment lengths, confirming the presence of these resistance determinants in the isolate.

### 3.9. Pathogenicity Assessment of the Isolate in Mice

#### 3.9.1. Determination Results of Median Lethal Dose (LD_50_)

Colony enumeration showed that plates inoculated with the 10^−8^ dilution met the criteria for viable bacterial counting, yielding 61, 67, and 55 colonies, respectively. Based on these counts, the concentration of the original bacterial suspension was calculated to be 6.1 × 10^9^ CFU/mL.

Mice inoculated with bacterial suspensions at concentrations of 10^10^ CFU/mL and 10^9^ CFU/mL exhibited obvious clinical signs within 24 h post-infection, including ruffled fur, lethargy, closed eyes, increased periocular secretions, markedly reduced feed intake, and mortality. In contrast, mice challenged with lower bacterial doses developed only mild clinical symptoms, and no deaths were observed in these groups. Mortality was recorded over a 7-day observation period, and the results are summarized in Table 5.

Based on the mortality data presented in Table 5, the median lethal dose (LD_50_) of the isolate was calculated using the Reed–Muench method and was determined to be 1.37 × 10^8^ CFU/mL. Mortality was observed primarily in mice challenged with relatively high bacterial concentrations. These results indicate that the isolate can cause lethal systemic effects in mice following high-dose intraperitoneal inoculation. However, this finding reflects experimental pathogenic potential in the murine model and should not be interpreted as evidence of pathogenicity in cattle.

#### 3.9.2. Observation Results of Tissue Lesions in Infected Mice

**Figure 10:**
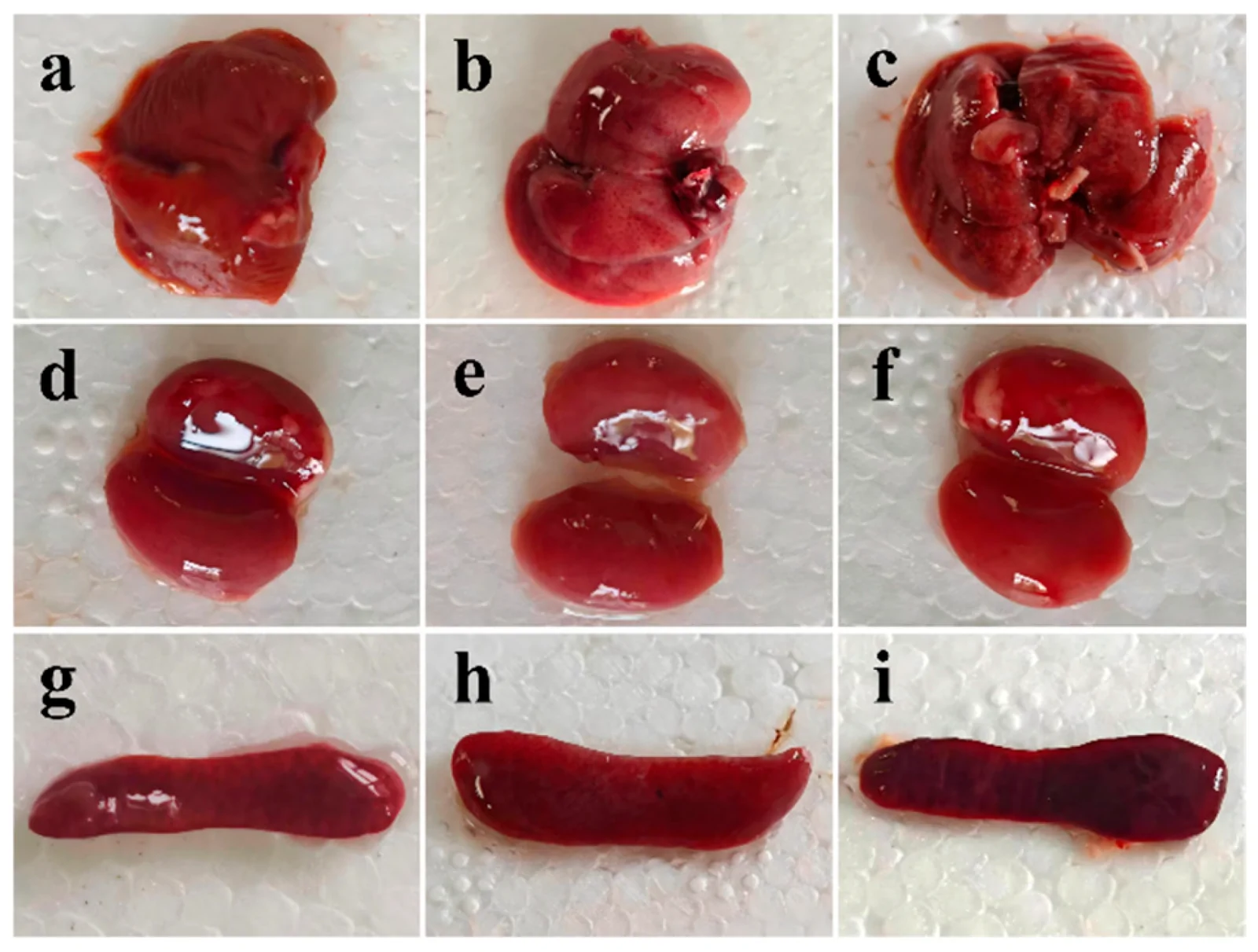
Gross anatomical lesions in tissues from mice (**a**) Ctrl, Liver; (**b**) OCI, Liver; (**c**) 10 × CI, Liver; (**d**) Ctrl, Kidney; (**e**) OCI, Kidney; (**f**) 10 × CI, Kidney; (**g**) Ctrl, Spleen; (**h**) OCI, Spleen; (**i**) 10 × CI, Spleen. Abbreviations: Ctrl = Control group; OCI = Original concentration infection group; 10 × CI = 10-fold concentrated infection group.

Gross examination revealed extensive pathological lesions in multiple organs of infected mice. The liver was markedly enlarged with rounded margins and exhibited a dark reddish-purple discoloration. Multiple grayish-white necrotic foci, ranging from pinpoint to millet-seed size, as well as scattered petechial hemorrhages, were observed on the hepatic surface. The liver parenchyma appeared friable and fragile. The kidneys showed tense capsules that were readily detachable and displayed a dark red appearance with diffuse congestion and scattered petechial hemorrhages on the surface. The spleen was markedly enlarged and dark purple in color, with a firm consistency and multifocal areas of congestion visible on the splenic surface (Figure 10).

**Figure 11:**
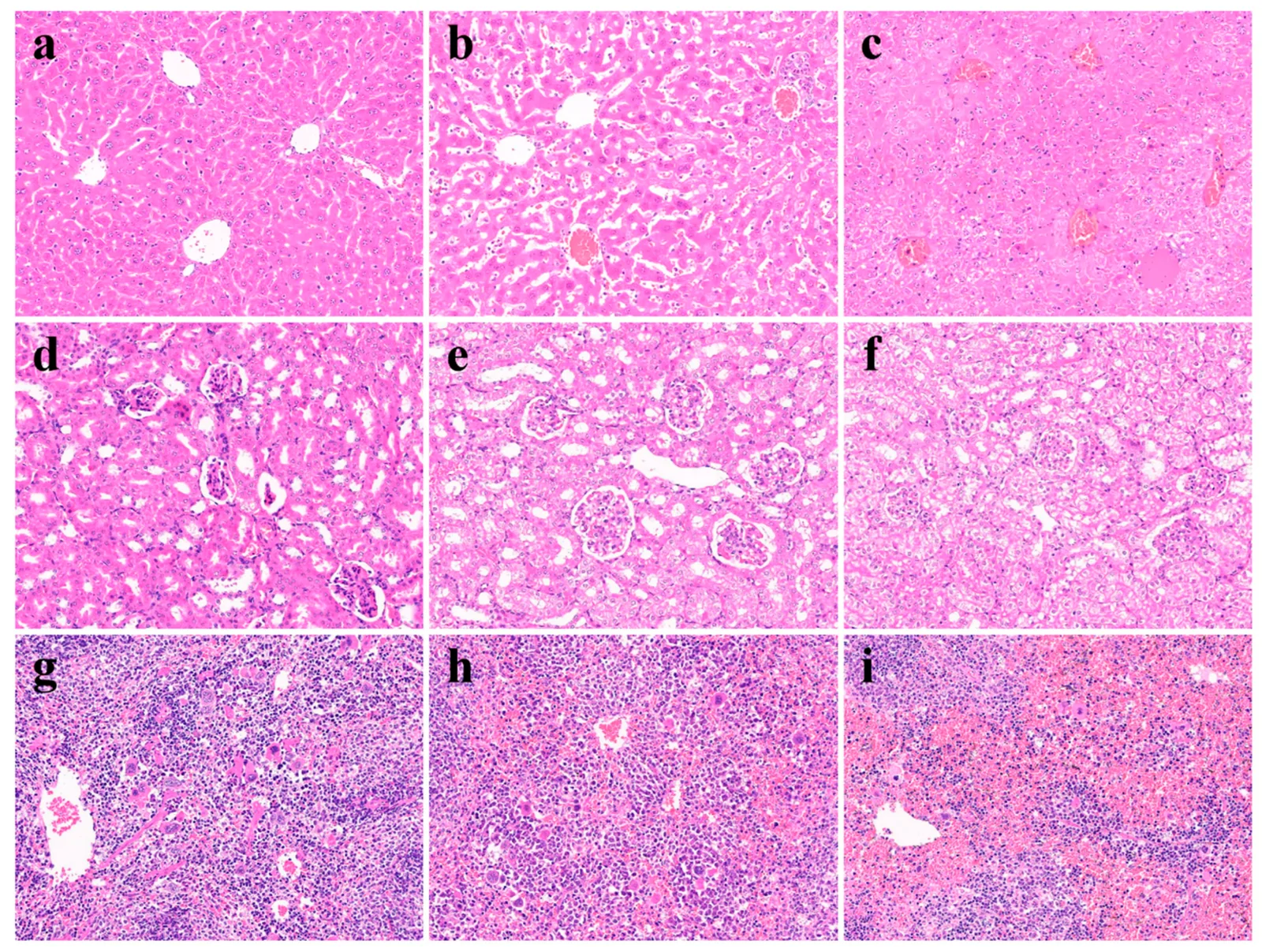
Histopathological examination of tissues from mice (20.0×) (**a**) Ctrl, Liver; (**b**) OCI, Liver; (**c**) 10 × CI, Liver; (**d**) Ctrl, Kidney; (**e**) OCI, Kidney; (**f**) 10 × CI, Kidney; (**g**) Ctrl, Spleen; (**h**) OCI, Spleen; (**i**) 10 × CI, Spleen. Abbreviations: Ctrl = Control group; OCI = Original concentration infection group; 10 × CI = 10-fold concentrated infection group.

Histopathological examination further confirmed progressive tissue damage in infected mice (Figure 11). In the control group, the hepatic lobular architecture was intact, hepatocytes were regularly arranged, and no evidence of congestion, inflammation, or necrosis was observed (Figure 11a). Mild lesions were detected in the low-dose infection group, characterized by slight sinusoidal dilation and congestion, focal hepatocellular swelling, and limited inflammatory cell infiltration within the portal areas (Figure 11b). In contrast, severe hepatic lesions were observed in the high-dose infection group, including extensive congestion and hemorrhage throughout the hepatic parenchyma, marked hepatocellular swelling and ballooning degeneration, multifocal necrosis, and diffuse infiltration of large numbers of neutrophils and lymphocytes (Figure 11c).

Normal renal histology was observed in control mice, with intact glomerular and tubular structures and no apparent interstitial abnormalities (Figure 11d). Mild pathological changes in infected mice included slight edema of proximal tubular epithelial cells and sparse inflammatory cell infiltration within the renal interstitium (Figure 11e). Severe renal injury was characterized by extensive necrosis and desquamation of renal tubular epithelial cells, accumulation of proteinaceous casts and cellular debris within tubular lumina, marked interstitial edema, diffuse inflammatory cell infiltration, and mild glomerular congestion (Figure 11f).

The spleens of control mice exhibited distinct white and red pulp regions with normal lymphoid follicular architecture (Figure 11g). In mildly affected animals, reactive hyperplasia of the white pulp and enlargement of lymphoid follicles were evident, accompanied by mild congestion of the red pulp (Figure 11h). In severely affected mice, the boundary between the white and red pulp was completely obscured, with marked white pulp atrophy and disruption of lymphoid follicular architecture. Additionally, severe congestion and hemorrhage were observed within the red pulp, together with multifocal coagulative necrosis and small abscess-like lesions (Figure 11i).

Collectively, these findings demonstrate that the bovine-derived *Providencia stuartii* isolate induced pronounced pathological alterations in the liver, kidney, and spleen, indicating its capacity to disseminate systemically and cause substantial tissue injury in experimentally infected mice.

#### 3.9.3. Bacterial Loads in Organs of Infected Mice

Quantitative bacterial culture demonstrated successful colonization of multiple organs following experimental infection. For kidney samples, plates from the 10^−5^ dilution met the criteria for colony enumeration, yielding 68 and 63 colonies, respectively. The bacterial burden in the kidney was calculated to be 5.90 × 10^8^ CFU/g. For liver samples, plates from the 10^−5^ dilution produced 112 and 111 colonies, corresponding to a bacterial load of 1.00 × 10^9^ CFU/g. In spleen samples, the 10^−6^ dilution plates were suitable for enumeration, with colony counts of 129 and 133, resulting in a calculated bacterial burden of 1.18 × 10^10^ CFU/g.

Among the examined organs, the spleen harbored the highest bacterial load, followed by the liver and kidney. The markedly elevated bacterial burden in the spleen suggests extensive bacterial proliferation within the reticuloendothelial system and is consistent with the severe splenic lesions observed histopathologically. These results indicate that the bovine-derived *P. stuartii* strain is capable of efficient systemic dissemination and persistent colonization of internal organs, thereby contributing to the development of severe pathological damage in infected mice.

## 4. Discussion

In the present study, the isolate was obtained from a diarrheic beef cattle individual, suggesting a possible association between *P. stuartii* carriage and enteric disorders. However, isolation from diarrheic animals alone does not establish a causal relationship between the organism and disease development. Diarrhea in cattle is frequently multifactorial and may involve viral, bacterial, parasitic, nutritional, and environmental factors. Therefore, the isolate should be considered a diarrhea-associated *P. stuartii* strain rather than a confirmed causative agent of bovine diarrhea. Therefore, the isolate should be considered a cattle-associated, diarrhea-associated *P. stuartii* isolate rather than a confirmed causative agent of bovine diarrhea. More importantly, its recovery highlights the presence of antimicrobial-resistant *P. stuartii* within the cattle production environment.

*Providencia stuartii* is an opportunistic pathogen of considerable clinical importance within the order Enterobacterales. It can exist as a commensal inhabitant of the intestinal tract or environmental niches but may cause infection when host immunity is compromised, antimicrobial selection pressure increases, or epithelial barriers are disrupted [1,17,18,19]. Previous studies have primarily focused on human clinical infections, particularly catheter-associated urinary tract infections, bacteremia, and healthcare-associated infections in hospitalized patients [1,2,3,17,18,19]. In contrast, information regarding the epidemiology, virulence characteristics, and antimicrobial resistance mechanisms of animal-derived *P. stuartii* remains relatively limited. The recovery of this organism from cattle suggests that livestock may represent one ecological niche for antimicrobial-resistant *P. stuartii*. From a One Health perspective, intestinal carriage in food-producing animals is important because resistant bacteria may be shed through feces into manure, wastewater, bedding materials, and surrounding farm environments, thereby contributing to the persistence and circulation of antimicrobial resistance within interconnected animal–environment ecosystems.

Genomic characterization revealed that the genome size and GC content of the isolate were comparable to those reported for the reference strain *P. stuartii* ATCC 33672 [20]. Comparative genomic analyses have demonstrated substantial diversity among *Providencia* species in genes associated with nutrient transport, energy metabolism, antimicrobial resistance, and environmental adaptation, with *P. stuartii* and *P. rettgeri* generally exhibiting enhanced adaptability to diverse ecological niches [21]. Consistent with these findings, the isolate examined in this study possessed a large number of genes involved in metabolism, membrane transport, signal transduction, and genetic information processing, suggesting a robust genetic basis for survival under intestinal, environmental, and stress-related conditions. Furthermore, multiple genomic elements, including CRISPR regions, prophages, genomic islands, and transposable elements, were identified, indicating considerable genomic plasticity. Mobile genetic elements are recognized as major vehicles for the acquisition and dissemination of antimicrobial resistance and virulence genes in members of the Enterobacteriaceae [22,23,24]. Although these genomic features are commonly associated with genome evolution and genetic exchange in Enterobacterales, the present study did not determine whether the detected antimicrobial resistance genes are located on transferable mobile genetic elements. Therefore, horizontal gene transfer cannot be inferred directly from the current data. Nevertheless, these genomic characteristics suggest that the isolate warrants further investigation regarding its potential role as a reservoir of antimicrobial resistance determinants.

Although *P. stuartii* is generally regarded as less virulent than many other enterobacterial pathogens, accumulating evidence suggests that it can contribute to disease through mechanisms involving adhesion, motility, invasion, biofilm formation, and iron acquisition [10,24]. The isolate characterized in this study harbored several virulence-associated genes, including *mrk*A, *fpt*A, *ire*A, *iut*A, and *hly*A. The *mrk*A gene is associated with fimbrial-mediated adhesion and colonization, whereas the iron acquisition genes *fpt*A, *ire*A, and *iut*A facilitate bacterial survival and competitiveness under iron-limited conditions encountered within the host. The *hly*A gene has been implicated in host cell damage and inflammatory responses [22,23,24,25,26,27,28]. Notably, despite the presence of *hly*A, no obvious hemolytic activity was observed on blood agar plates. This discrepancy highlights that the presence of a virulence gene does not necessarily translate into phenotypic expression. Gene expression may be influenced by culture conditions, regulatory networks, gene integrity, and host-specific environmental factors. Consequently, additional investigations, including cell adhesion and invasion assays, hemolytic activity measurements, transcriptional analyses, and in vivo infection studies, are required to verify the functional roles of these virulence determinants. From a One Health perspective, the presence of multiple antimicrobial resistance determinants in a cattle-associated *P. stuartii* isolate is of particular concern. Livestock-associated bacteria carrying resistance genes may contribute to the maintenance of antimicrobial resistance within animal production systems. Following fecal excretion, resistant bacteria may enter manure, wastewater, soil, surface water, and other environmental compartments, where opportunities for interaction with diverse bacterial communities may arise. Although the present study did not investigate environmental dissemination or horizontal gene transfer experimentally, these findings support the inclusion of *P. stuartii* in integrated antimicrobial resistance surveillance programs encompassing livestock, farm environments, food production systems, and human health.

Antimicrobial resistance represents one of the most concerning biological characteristics of *P. stuartii*. Clinical studies have shown that this species frequently exhibits multidrug-resistant phenotypes and may produce extended-spectrum β-lactamases (ESBLs), AmpC β-lactamases, and even carbapenemases, thereby posing significant therapeutic challenges [2,4,19,29,30,31]. In the present study, the isolate exhibited resistance or reduced susceptibility to multiple classes of antimicrobial agents and carried several resistance genes, including *bla*_CTX-M_, *aad*A1, *sul*1, and *tet*A. These findings indicate a genetic basis for resistance to β-lactams, aminoglycosides, sulfonamides, and tetracyclines. Among these determinants, *bla*_CTX-M_ is one of the most prevalent ESBL genotypes circulating among Enterobacteriaceae worldwide and is frequently associated with plasmids, integrons, and transposons [22,23,24,29]. Likewise, *aad*A1, *sul*1, and *tet*A are commonly identified in multidrug-resistant bacterial isolates and often participate in the formation and horizontal transfer of resistance gene clusters [22,23,24]. Some discrepancies between genotypic and phenotypic resistance profiles were observed in this study, which may be attributable to differences in gene expression levels, outer membrane permeability, efflux pump activity, enzyme variants, and limitations inherent to disk diffusion susceptibility testing [32,33,34]. Therefore, comprehensive assessment of antimicrobial resistance in animal-derived *P. stuartii* should integrate phenotypic susceptibility testing, molecular detection of resistance genes, and whole-genome analyses to avoid overreliance on any single method. Accordingly, the presence of virulence-associated genes should be interpreted as evidence of potential biological capability rather than confirmed pathogenicity.

Previous studies have reported the isolation of *P. stuartii* from diarrheic calves [8] and its involvement in fatal septicemia in rhesus macaques [9], suggesting that this bacterium should not be regarded merely as a transient commensal or environmental contaminant in animals. Considering the virulence genes, resistance determinants, and pathogenicity observed in the present study, it is reasonable to speculate that this strain may contribute to intestinal infections, secondary bacterial infections, or septicemic conditions in cattle under specific circumstances. Nevertheless, because the isolate was recovered from rectal swabs rather than diseased tissues, its role as a primary pathogen cannot be conclusively established. Its virulence and resistance potential, however, indicate that *P. stuartii* and other uncommon opportunistic pathogens should be included in routine etiological surveillance programs for cases of diarrhea, septicemia, or unexplained infections in intensive cattle production systems. The murine infection results should be interpreted within the limitations of the experimental model. Intraperitoneal inoculation bypasses the intestinal mucosal barrier and directly exposes the peritoneal cavity and internal tissues to a large bacterial inoculum. This route does not represent the presumed natural exposure route of cattle-associated *P. stuartii*. In addition, mortality was mainly observed at relatively high challenge concentrations, and such doses may not reflect bacterial exposure levels encountered under field conditions. Host-species differences in innate immunity, tissue susceptibility, bacterial receptor distribution, and microbiota composition may also influence the outcome of infection. Therefore, systemic dissemination, organ bacterial burdens, and pathological lesions observed in mice cannot be extrapolated as evidence that the isolate can invade bovine tissues or cause disease in cattle. Despite these limitations, the murine model provided preliminary biological evidence that the genomic virulence-associated determinants identified in the isolate were accompanied by measurable effects in vivo. The model allowed evaluation of dose-dependent mortality, systemic recovery of the organism, and tissue injury under standardized conditions. Accordingly, the experiment should be regarded as an initial screening assay of pathogenic potential rather than confirmation of bovine pathogenicity. Establishing a causal role in bovine diarrhea would require additional evidence, including repeated isolation from clinically affected animals, comparison with healthy controls, exclusion of major enteric pathogens, demonstration of bacterial localization in bovine lesions, and, where ethically and practically feasible, challenge studies using cattle-relevant exposure routes.

From a public health perspective, livestock farms act as critical reservoirs of antimicrobial-resistant bacteria and resistance genes via animal intestinal tracts, manure, wastewater, and bedding materials. Given its robust environmental adaptability and ability to capture resistance determinants via mobile genetic elements [21,22,23,24], the primary implication of this isolate is its potential function as a resistance reservoir within livestock-related ecosystems, rather than confirming its pathogenicity in cattle. Food animals are widely acknowledged as a vital component of the One Health framework. Antimicrobial-resistant bacteria colonizing the intestinal tract can be continuously excreted into manure, wastewater, bedding, and surrounding environments. These ecological interfaces sustain resistant bacterial populations and enable genetic exchange with other Enterobacterales species. Clinical medication regimens should be formulated based on antimicrobial susceptibility testing wherever feasible. Excessive empirical administration of broad-spectrum antimicrobials ought to be restricted to alleviate the selective pressure that drives the proliferation of multidrug-resistant strains [12,13,35,36].

Several limitations of the present study should be acknowledged. First, the isolate originated from a single farm, and the sample size was limited, preventing broader conclusions regarding the epidemiology of bovine-derived *P. stuartii*. Second, virulence and resistance determinants were identified primarily through PCR assays and bioinformatic predictions, without validation at the transcriptional, protein, or functional levels. Third, although the murine intraperitoneal infection model provides valuable information regarding systemic pathogenicity, it does not fully replicate the natural route of infection in cattle. In addition, environmental samples, manure, wastewater, healthy cattle, and human-associated isolates were not included, preventing assessment of the ecological distribution of the organism and possible transmission pathways within the One Health continuum. Furthermore, long-read genome sequencing and experimental horizontal gene transfer assays were not performed, precluding evaluation of the genomic mobility of the detected resistance determinants. Future studies should include larger sample collections and a greater number of isolates, combined with multilocus sequence typing, pangenome analysis, plasmid sequencing, cell infection models, and targeted functional studies to comprehensively elucidate the transmission dynamics, pathogenic mechanisms, and antimicrobial resistance dissemination potential of bovine-derived *P. stuartii*.

## 5. Conclusions

In this study, a cattle-associated *Providencia stuartii* isolate carrying multiple antimicrobial resistance determinants was recovered from a rectal swab of diarrheic beef cattle and characterized using phenotypic, molecular, and whole-genome approaches. The isolate exhibited resistance or reduced susceptibility to multiple antimicrobial classes and possessed diverse resistance-associated genes together with genomic features indicative of substantial genome plasticity.

The principal significance of this isolate lies in its potential role as a reservoir of antimicrobial resistance within livestock-associated ecosystems rather than as a confirmed bovine pathogen. Recovery of *P. stuartii* from cattle highlights the importance of including uncommon opportunistic bacteria in integrated One Health surveillance involving livestock, manure, wastewater, farm environments, food-production systems, and human-associated settings.

Although high-dose intraperitoneal inoculation demonstrated experimental pathogenic potential in mice, these findings do not establish natural pathogenicity, tissue invasion, or disease causation in cattle. Likewise, while the isolate carried multiple antimicrobial resistance determinants, the mobility and horizontal transfer potential of these genes were not experimentally evaluated and therefore remain to be determined. Future studies integrating epidemiological surveillance, environmental sampling, complete genome sequencing, and horizontal gene transfer analyses will be essential to clarify the ecological significance of cattle-associated *P. stuartii* and its contribution to antimicrobial resistance dissemination within the One Health framework.

## Figures and Tables

**Table 1:** Primer information of virulence genes in *P. stuartii*.

Genes		Oligonucleotide Sequence	Fragment Length (bp)
Adhesins	*fim*A	F: GCATGTGCTGTTGATGCTAAC	215
		R: GAACCCGCAACAGCTAGAA	
	*mrk*A	F: GCCAAGGTAGTGACGCTAAA	411
		R: TTTCTACGATACCGCTGCTAAC	
Siderophores	*fpt*A	F: ACTGCTCCATGGGCTTTATC	744
		R: CATAGGTACGCCAGCCATATT	
	*ire*A	F: CCCGGCGAATACACCTTAAT	351
		R: GTAATCGCCACCACCATACA	
	*iut*A	F: TCTGGCGCGACTTCTTTATATG	347
		R: GTGGCTTGTAGCTGCTGATTA	
Hemolysin	*hly*A	F: TCGCCATCTTAGCAGGTATTG	701
		R: AGCTTCGAGTGTATCGGAAAG	

**Table 2:** Primer information of antimicrobial resistance genes in *P. stuartii*.

Genes		Oligonucleotide Sequence	Fragment Length (bp)
β-lactams	*bal*_TEM-1_	F: TTCGTGTCGCCCTTATTC	820
		R: CCTATCTCAGCGATCTGTCTA	
	*bla*_CTX-M_	F: CGATAACGTGGCGATGAA	416
		R: TAGGTGAGGCTGGGTGA	
aminoglycoside	*aac*3	F: GGACACGATGCCAACACG	200
		R: CCGACTGGACCTTCCTTCTG	
	*aad*A1	F: GTGGATGGCGGCCTGAAGCC	527
		R: ATTGCCCAGTCGGCAGCG	
sulfonamide	*sul*1	F: ACGTATTGCGCCGCTCTT	555
		R: TGGGTGCGGACGTAGTCA	
tetracycline	*tet*A	F: CAGGCAGGTGGATGAGGAAC	175
		R: CGGCAGGCAGAGCAAGTAG	
chloramphenicol	*cat*	F: ATCCCAATGGCATCGTAA	209
		R: CCAGCTCACGGTCTTCTA	

**Table 3:** Results of biochemical characterization.

Biochemical Test	Result	Biochemical Test	Result
Semi-solid agar Ornithine decarboxylase broth	+ −	Methyl red Phenylalanine deaminase	+ +
Lysine decarboxylase broth	−	Mannitol	−
Amino acid decarboxylase control	−	Inositol	+
Simmons citrate agar	+	Sorbitol	−
H_2_S	−	Melibiose	−
Urease	−	Ribitol	−
Indole production	+	Raffinose	−

Notes: + indicates positive reaction; − indicates negative reaction.

**Table 4:** Drug susceptibility test results of the *P. stuartii* isolate.

Antimicrobial Agent	Zone (mm)	Antimicrobial Agent	Zone (mm)
Gentamicin	7	Cefazolin	12
Kanamycin	9	Cefoperazone	21
Amikacin	3	Norfloxacin	17
Tetracycline	10	Ciprofloxacin	17
Minocycline	10	Ofloxacin	19
Ampicillin	14	Trimethoprim- sulfamethoxazole	15
Ceftazidime	20		
Ceftriaxone	22	Erythromycin	0

**Table 5:** Determination results of the median lethal dose (LD_50_) in mice.

Inoculum Dose (CFU/mL)	Number of Diseased Mice	Morbidity (%)	Number of Dead Mice	Mortality (%)
10^10^	8	100	8	100
10^9^	7	87.5	5	62.5
10^8^	1	12.5	1	12.5
10^7^	0	0	0	0
10^6^	0	0	0	0

## Data Availability

All data generated or analyzed during this study are included in this published article.
